# Low Intensity Repetitive Transcranial Magnetic Stimulation Does Not Induce Cell Survival or Regeneration in a Mouse Optic Nerve Crush Model

**DOI:** 10.1371/journal.pone.0126949

**Published:** 2015-05-20

**Authors:** Alexander D. Tang, Kalina Makowiecki, Carole Bartlett, Jennifer Rodger

**Affiliations:** Department of Experimental and Regenerative Neurosciences, School of Animal Biology, The University of Western Australia, Perth, WA, Australia; Universidade Federal do Rio de Janeiro, BRAZIL

## Abstract

Low intensity repetitive Transcranial Magnetic Stimulation (LI-rTMS), a non-invasive form of brain stimulation, has been shown to induce structural and functional brain plasticity, including short distance axonal sprouting. However, the potential for LI-rTMS to promote axonal regeneration following neurotrauma has not been investigated. This study examined the effect of LI-rTMS on retinal ganglion cell (RGC) survival, axon regeneration and levels of BDNF in an optic nerve crush neurotrauma model. Adult C57Bl/6J mice received a unilateral intraorbital optic nerve crush. Mice received 10 minutes of sham (handling control without stimulation) (n=6) or LI-rTMS (n = 8) daily stimulation for 14 days to the operated eye. Immunohistochemistry was used to assess RGC survival (β-3 Tubulin) and axon regeneration across the injury (GAP43). Additionally, BDNF expression was quantified in a separate cohort by ELISA in the retina and optic nerve of injured (optic nerve crush) (sham n = 5, LI-rTMS n = 5) and non-injured mice (sham n = 5, LI-rTMS n = 5) that received daily stimulation as above for 7 days. Following 14 days of LI-rTMS there was no significant difference in mean RGC survival between sham and treated animals (p>0.05). Also, neither sham nor LI-rTMS animals showed GAP43 positive labelling in the optic nerve, indicating that regeneration did not occur. At 1 week, there was no significant difference in BDNF levels in the retina or optic nerves between sham and LI-rTMS in injured or non-injured mice (p>0.05). Although LI-rTMS has been shown to induce structural and molecular plasticity in the visual system and cerebellum, our results suggest LI-rTMS does not induce neuroprotection or regeneration following a complete optic nerve crush. These results help define the therapeutic capacity and limitations of LI-rTMS in the treatment of neurotrauma.

## Introduction

Non-invasive brain stimulation can be used to modulate neural activity in the central (CNS) and peripheral nervous systems (PNS) and has been applied in diagnosis and treatment of neurological disorders. One form of non-invasive brain stimulation is repetitive transcranial magnetic stimulation (rTMS), in which time-varying magnetic pulses from a coil placed over the skull induce electrical currents in the underlying brain by Faraday Induction. rTMS is used clinically at high and low intensities in a wide range of neurological and psychiatric conditions, with therapeutic effects that can persist for hours to days after stimulation [1–4].

The best-characterised effects of rTMS in human patients are alterations in cortical excitability that persist beyond the time of stimulation [5, 6]. Mechanisms underpinning these effects have been explored in animal models and demonstrate altered synaptic plasticity in the form of long-term potentiation [7, 8]. Furthermore, functional imaging of human patients suggests that repeated rTMS delivery may trigger structural and functional reorganisation [9] and our recent work in mice has confirmed structural and functional reorganisation of abnormal brain circuits via removal or shifting of inappropriate connections, even using low intensity magnetic stimulation (LI-rTMS) (12mT field strength) [10, 11].

Whilst the biological mechanisms of rTMS are poorly defined, a key molecule up-regulated by both rTMS and LI-rTMS effects is brain derived neurotrophic factor (BDNF) [10–13], a powerful and versatile signalling molecule that plays many roles not only in synaptic plasticity, but also in promoting neuronal survival and axonal outgrowth. Furthermore, delivery of exogenous BDNF either by viral overexpression or injection of recombinant protein showed neuroprotective and neuroregenerative effects in a range of CNS injury models [14–16]. We thus hypothesised that LI-rTMS may be useful in promoting cell survival and/or axonal regeneration following brain injury, via up-regulation of BDNF. In agreement with this hypothesis, there is some indication that rTMS may promote neuronal survival in the lesion site following an ischaemic stroke [17], and studies in the PNS show that direct electrical stimulation can promote regeneration following nerve damage [18, 19]. However, the use of rTMS as a neuroprotective and/or neuroregenerative intervention following neurotrauma has not been well characterised.

Here we investigate the effects of LI-rTMS on neuronal survival and axonal regeneration using a complete optic nerve crush model. The optic nerve is a white matter tract, consisting of axons from a single cell type in the retina, the retinal ganglion cell (RGC). The absence of any surrounding gray matter allows for the investigation of cell survival and neuronal regeneration as distinct events following injury [19]. In addition, optic nerve injury models have been used extensively to investigate the potential of both neurotrophin [19–21] and electrical stimulation treatments on cell survival and regeneration [22–25]. Therefore the optic nerve crush provides an ideal model to investigate the efficacy of LI-rTMS in neuroprotection and regeneration. We delivered LI-rTMS for 10 minutes daily for 14 days at a high frequency biomimetic pattern to the left eye of C57Bl/6J mice starting from the day after optic nerve crush. We chose this protocol because we have previously shown that it induces plastic reorganisation and robust up-regulation of BDNF expression in the mouse visual cortex, superior colliculus [10, 11] and cerebellum [26]. Here, we found that LI-rTMS had no effect on RGC survival or axonal regeneration, and that BDNF expression was not up regulated in the retina or optic nerve of non-injured and injured mice (optic nerve crush). Our findings suggest that different brain regions may respond differently to LI-rTMS in terms of BDNF up-regulation, and that the therapeutic applications of LI-rTMS will need to be tested in a range of models in order to establish the scope and limitations of this technique.

## Methods

### Animals

3 month old C57Bl /6J mice (*Mus Musculus*) (n = 34) (Animal Research Centre, Murdoch University, WA, Australia) were group housed on a 12-hour light/dark cycle with food and water *ad libitum*. All animal work was conducted according to Australian and international guidelines. Mice were euthanased with an overdose of pentobabitone (>160mg/kg i.p.) and anaesthetised with ketamine and medetomidine (75 and 1 mg/kg respectively i.p.).

Procedures were approved by the Animal Ethics Committee of the University of Western Australia (approval id: RA100/03/1214).

### Optic nerve crush

Surgery was performed on the left eye to obtain a complete unilateral optic nerve crush. Mice were anaesthetised with an intraperitoneal injection of ketamine and medetomidine (75 and 1 mg/kg respectively, Troy Ilium, NSW, Australia). An incision to the lateral conjunctiva allowed for slight rotation of the globe. Muscle and connective tissue were gently separated to expose the optic nerve. The exposed nerve was crushed using Dumont #5 forceps (World Precision Instruments, FL, USA) for 5 seconds, 2-3mm from the optic nerve head. Forceps were gently removed, allowing the eye to rotate back into place. Anaesthesia was reversed with subcutaneous injection of atipamezole (1 mg/kg, Troy Ilium, NSW, Australia). We confirmed no surviving axons in the nerve distal to the lesion using CTB and beta tubulin in a small number of animals (data not shown).

The contralateral (non-injured) retina was **not** used for control tissue, as unilateral optic nerve crush can induce bilateral glial cell activation [27, 28] and RGC loss [29, 30].

### LI-rTMS

**Fig 1:**
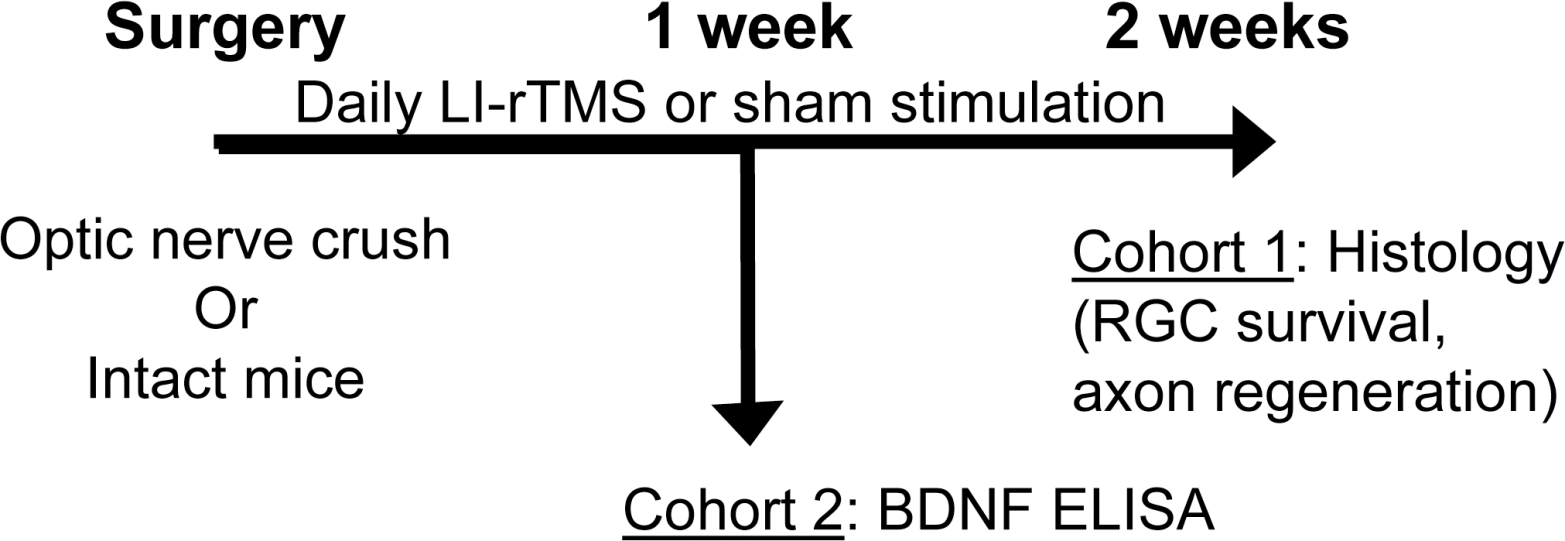
Diagrammatic representation of the study design. Mice received an optic nerve crush and were separated into two cohorts for (i) daily LI-rTMS or sham stimulation and assessed for RGC survival and axonal regeneration (2 weeks survival) (ii) quantification of BDNF levels by ELISA (1 week survival). With the second cohort, additional control groups of intact mice with no optic nerve crush were processed in parallel for BDNF analysis with the same LI-rTMS or sham stimulation parameters.

LI-rTMS or sham (handling control without stimulation) was delivered daily to the operated eye for 10 minutes. Mice were randomised into two cohorts (Fig 1). In cohort 1 (n = 14), mice received an optic nerve crush and 14 days of stimulation (sham n = 6, LI-rTMS n = 8) to assess RGC survival and axonal regeneration. In cohort 2 (n = 20), mice received 7 days of stimulation to assess changes in BDNF concentrations with or without an optic nerve crush. Such that there were 5 animals per group (LI-rTMS + optic nerve crush, sham + optic nerve crush, LI-rTMS without optic nerve crush and sham without optic nerve crush). An electromagnetic pulse generator (Global Energy Medicine, WA, Australia) delivered a high frequency complex pattern of stimulation consisting of 59.9ms trains of 20 pulses with trains repeated at 6.67Hz for the first minute, 10.01Hz for 8 minutes and then 6.25Hz for 1 minute. This protocol was selected as it mimics endogenous patterns of electrical activity in the nervous system (patent PCT/AU 2007/00045) and has been shown to up-regulate BDNF and facilitate circuit reorganisation in the visual system[10, 11] and cerebellum [26].

A custom-made coil (8mm outer diameter, consisting of 300 windings of 0.25mm copper wire, with a steel bolt core, 16Ω) was used to deliver LI-rTMS [10] to the operated eye. Magnetic field strength was measured with a Hall Effect probe (Honeywell SS94A2D, USA). Magnetic field strength decreased with distance from 12mT at the base of the coil to 1.8mT at a distance of 8mm from the coil base. Therefore we estimate the magnetic field strength in the orbital area ranged from 7.4 to 1.8mT. Conscious animals were stimulated under light manual restraint, to avoid possible confounding effects of anaesthetic as opposing effects of rTMS have been observed under anaesthesia (i.e. rTMS down-regulates BDNF in anaesthetised animals) [12]. Animals were placed head first into a small clear conical cylinder with a breathing hole at the end and were habituated to handling and restraint for a week prior to experimentation. Stimulation was delivered through the plastic cylinder, such that the coil was placed directly on the cylinder, immediately over the eye. We have previously shown the coils do not generate vibration [31] and the plastic does not impede the magnetic field.

### Tissue preparation

24 hours after the last stimulation, mice were terminally anaesthetised with 160mg/kg pentabarbitone sodium and transcardially perfused with 0.9% saline followed by 100mL of 4% paraformaldehyde in 0.1M phosphate buffer (pH 7.2). The left eye with optic nerve was dissected and separated at the optic head. Left eyes were enucleated and whole retinas dissected from the sclera. Retinas were post fixed for 12 hours in fixative solution and stored in PBS with 0.01% sodium azide prior to immunohistochemical analysis. Optic nerves were post fixed in fixative solution and transferred into 30% sucrose in PBS at 4°C for 48 hours. Optic nerves were embedded in optimum cutting temperature medium (Sakura, OH, USA) at -20°C. A Leica CM1900 cryostat was used to cut 14μm transverse sections that were thaw mounted onto gelatin coated glass slides for immunohistochemical analysis.

### Immunohistochemistry

RGC survival and axonal growth was evaluated in the retina and optic nerve, respectively. Retinas were processed free-floating and permeabilised with 0.2% Triton in 0.1M PBS followed by blocking with 10% donkey serum in 0.2% bovine serum albumin (Sigma Aldrich, MO, USA) in 0.1M PBS. Retinas were incubated with for β-3 tubulin primary antibody (1:1000 monoclonal mouse) (Merck Millipore, VIC, Australia) at 4°C for 24 hours. Following 3 rinses in PBS, retinas were incubated with Alexafluor-488 donkey anti-mouse IgG (Life Technologies, VIC, Australia) at room temperature for 4 hours. After three 10 minute washes in PBS, retinas were flattened onto glass slides and cover-slipped with Fluoromount-G (Sigma Aldrich, MO, USA).

Axonal regrowth of RGC axons was assessed in the optic nerve using growth associated protein 43 (GAP43) immunohistochemistry[32, 33]. Optic nerve sections were permeablised and blocked as described above and incubated with GAP43 primary antibody (1:1000 monoclonal mouse) (Merck Millipore, VIC, Australia) at 4°C for 24 hours. Following washes in PBS, optic nerve sections were incubated with Alexafluor-488 donkey anti-mouse IgG at room temperature for 4 hours, washed with PBS and cover-slipped as described above. GAP43 immunofluorescence was analysed with fluorescence microscopy (Nikon Eclipse 80i, 40x objective) (LMG Scientific Services, WA, Australia).

#### Stereological analysis of retinal wholemounts

Retinal wholemounts were analysed with the optical fractionator method to estimate the number of β-3 tubulin positive RGCs [34]. Wholemount outlines were digitised on a microscope (Olympus BX50, 10x objective), equipped with a motorised stage, and analysed with Stereoinvestigator software (MicroBrightField, VT, USA) (20x objective). RGCs were counted in a 150x150μm frame. Counting frames were placed systematically within a grid to achieve approximately 200 counting sites, covering approximately 25% of the total area of each retina. Extrapolated RGC populations were divided by retinal wholemount area and remaining RGCs (survival) expressed as RGC/mm^2^.

### Enzyme- Linked Immunosorbent Assay (ELISA) and Protein Assay

24 hours after the last stimulation, retinas and optic nerves were dissected from freshly euthanised mice (crush + sham, crush + LI-rTMS, non-injured + sham, non-injured + LI- rTMS) and stored at -80°C. Samples were homogenised in 1mL of lysis buffer[35] (100 mM PIPES pH 7, 500 mM NaCl, 0.2% Triton X-100, 2mM EDTA) with mini protease inhibitor tablets (Roche Biochemicals, IN, USA)(1 tablet added per 10ml buffer). Lysates were centrifuged (3320 x *g* at 4°C for 1 hour) to collect resulting supernatants. Supernatants were analysed by ELISA for BDNF as per manufacturer’s instructions (ChemiKine BDNF Sandwich ELISA, Chemicon International Inc., CA, USA). In addition, supernatants were analysed for total protein content (Pierce BCA Protein Assay Kit, Thermo Fisher Scientific, IL, USA, as per manufacturer’s instructions). Supernatant BDNF concentrations were normalised to supernatant total protein content for analyses and expressed as a percentage of the sham group for analyses.

### Statistical Analysis

Statistical analyses were performed with SPSS (version 20, IBM, NY, USA). Normal distribution and homogeneity of variance were verified before running parametric analyses. All means are presented with their respective standard error of the mean (i.e. mean ± SEM). Analyses were conducted on RGC survival (unpaired t-test) and BDNF concentrations (two-way between subjects ANOVA with Sidak corrected *post-hoc* tests). Results were classified as significant if p<0.05.

## Results

### LI-rTMS does not increase RGC survival

To investigate whether 14 days of LI-rTMS could induce neuroprotection and cell survival, we characterised the number of surviving RGCs at 15 days post optic nerve crush injury (Table 1). Normal intact C57Bl/6J mice have roughly 4000 RGCs per mm^2^ and cell counts suggest fewer than 10% of RGCs survived the optic nerve crush, with no significant difference in remaining RGCs between sham and LI-rTMS (t = (12) = 1.12, p = 0.284).

**Table 1:** RGC survival and BDNF concentrations following stimulation.

Group + stimulation	Tissue	RGC/mm^2^	BDNF % of total protein (x10^-5^)*
non-injured + sham	Retina	≈4000 [36]	1.26±0.05
	Optic Nerve	-	2.28±0.23
non-injured + LI-rTMS	Retina	-	1.34±0.008
	Optic Nerve	-	2.80±0.33
optic nerve crush + sham	Retina	237.2±60.33	1.37±.088
	Optic Nerve	-	2.97±0.34
optic nerve crush + LI-rTMS	Retina	335.4±60.74	1.53±0.12
	Optic Nerve	-	2.72±0.29

Mean (±SEM) RGC survival and BDNF concentrations following sham or LI-rTMS.

NOTE: RGC survival was quantified following 14 days of stimulation and BDNF was quantified following 7 days of stimulation (*).

### LI-rTMS does not induce RGC axon regeneration

**Fig 2:**
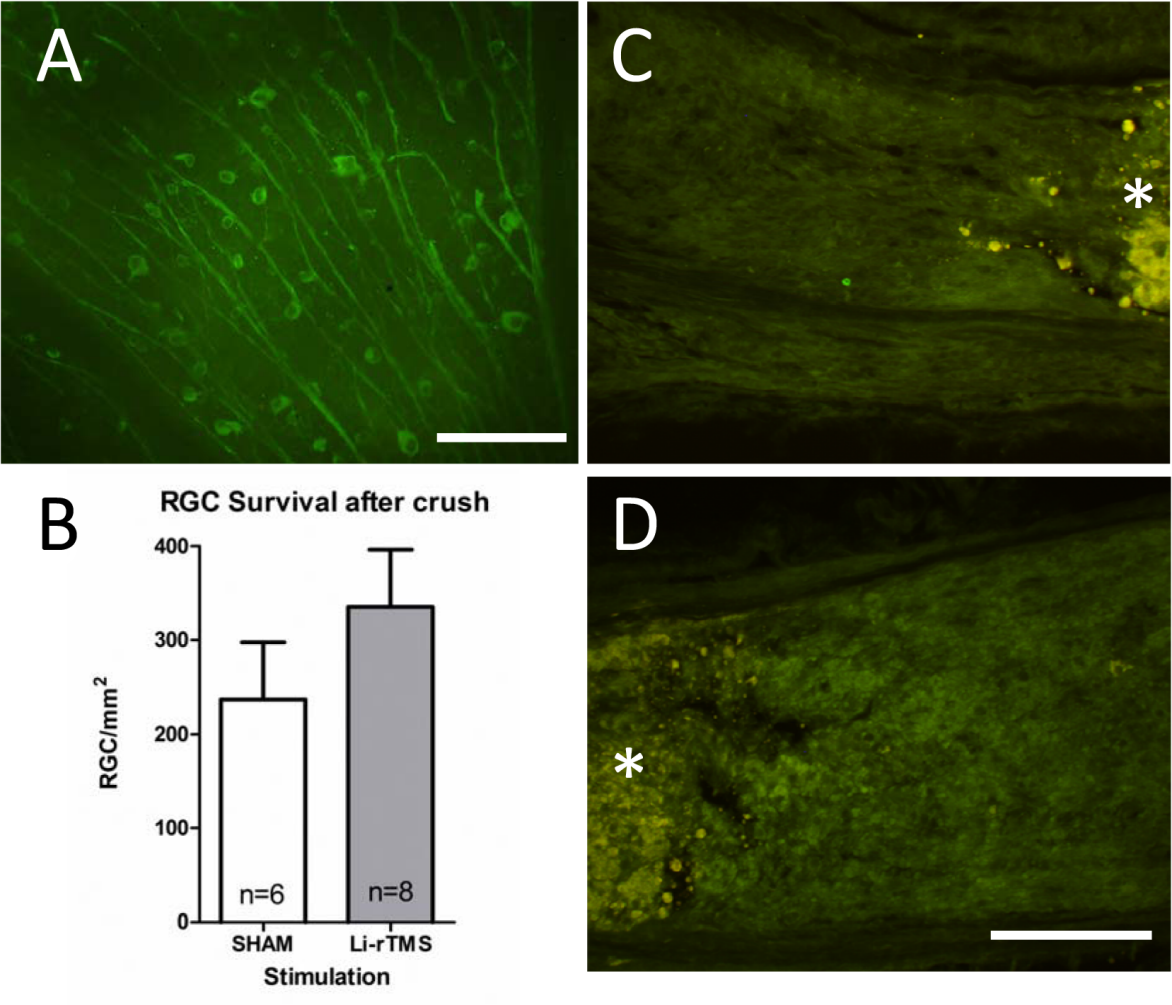
LI-rTMS does not affect RGC survival or axonal regeneration following optic nerve crush. A: photomicrograph showing RGCs immunolabelled with β3 tubulin following an optic nerve crush and 2 weeks of daily LI-rTMS. Scale bar is 100 μm. B: Histogram showing counts of surviving RGCs in LI-rTMS and sham stimulated retinas 2 weeks following an optic nerve crush. There was no significant difference between the stimulation groups (p = 0.256). Error bars are standard error of the mean. C, D: GAP-43 immunohistochemistry in the proximal (C) and distal (D) optic nerve did not result in labelling of any axons. The crush site is indicated by *. Scale bar 100 μm

To assess whether any regenerating RGC axons were present, transverse sections of the optic nerves were labelled using GAP43 immunohistochemistry (Fig 2). No GAP43 labelling was present on either side of the crush site in sham or LI-rTMS groups, indicating no axon regeneration distal or proximal to the crush site.

### LI-rTMS does not increase BDNF in the retina or optic nerve

**Fig 3:**
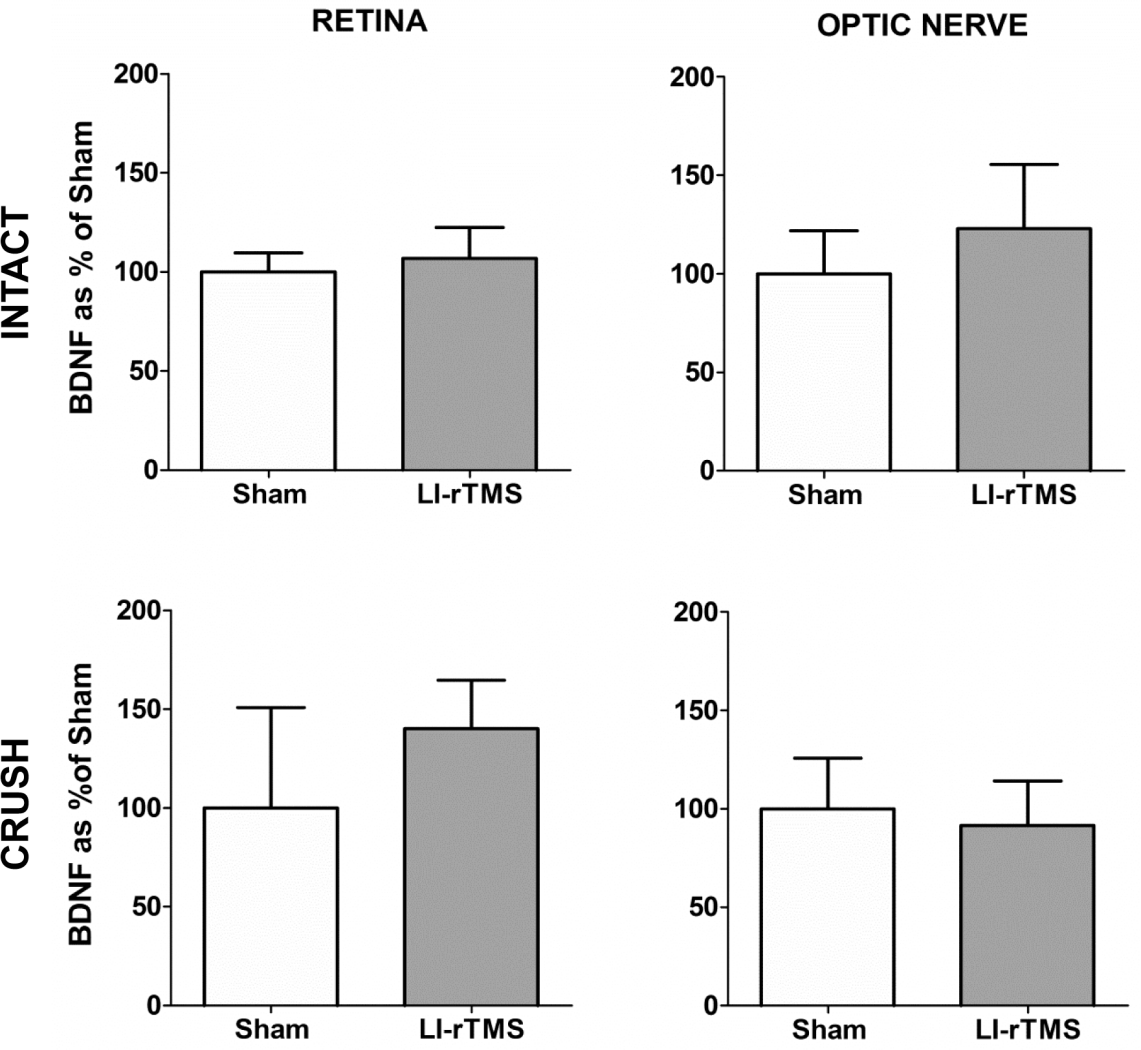
BDNF ELISA data. Daily LI-rTMS for 1 week does not increase BDNF levels in the retina or optic nerve of optic nerve crush or intact mice (p>0.05 for all groups; see results section). Histograms show BDNF levels as % of sham stimulated group. Error bars are standard error of the mean.

To investigate whether LI-rTMS up-regulates BDNF in intact and injured mice, retinas and optic nerves were collected for BDNF ELISA analysis (Table 1 and Fig 3). For retinal tissue, there was no significant difference between stimulation condition (sham vs. LI-rTMS) (F[1,16] = 1.919 p = 0.185) or between injury groups (non-injured vs. injured) (F[1,16] = 2.680, p = 0.121). Furthermore, there was no significant interaction between stimulation and injury conditions (F[1,16] = 0.209, p = 0.653). As we hypothesised *a priori* that LI-rTMS would up-regulate BDNF compared to sham, despite the non-significant main effect, we conducted Sidak-corrected *post-hocs*, restricted to comparisons between LI-rTMS and sham, which confirmed mean retinal BDNF concentrations were not significantly different between sham and LI-rTMS treated animals in either the non-injured (p = 0.771) and injured (p = 0.377) groups.

Similarly, in optic nerve tissue, there was no significant difference between stimulation conditions (F[1,16] = 0.199, p = 0.661) or injury groups (F[1,16] = 1.056, p = 0.319). There was no significant interaction between stimulation and injury conditions (F[1,16] = 1.626, p = 0.220). Follow up Sidak-corrected *post-hocs* confirmed no significant difference in the mean optic nerve BDNF concentrations between sham and LI-rTMS treated animals in the non-injured (p = 0.424) and injured (p = 0.820) groups.

## Discussion

Non-invasive brain stimulation techniques, and in particular rTMS, have become an increasingly common experimental treatment for neurological and psychiatric disorders. However, the extent to which rTMS can be used in the treatment of neurotrauma has not been well characterised. Our results show that, unlike in other visual brain regions examined previously (visual system:[10, 11]; cerebellum [26]), LI-rTMS did not up-regulate BDNF levels in either the intact or injured retina or in the optic nerve, and this was associated with a lack of pro-survival or pro-regenerative effects following injury. The implication is that different brain regions respond differently to LI-rTMS and it will be important to characterise these specific cellular and molecular responses in order to determine relevant and optimised use of electromagnetic stimulation for neural repair.

### Does intensity matter?

Typically, rTMS is delivered with commercial human sized coils using high intensity field strengths (≥1T) in both human and animal studies. However, in small animal studies, particularly rodents, a large coil to brain size ratio results in stimulation of the entire brain, if not the whole animal, reducing efficiency of the magnetic field [36]. This study delivered LI-rTMS through a custom coil (8mm outer diameter) which allowed for greater focality [10] at the expense of intensity (12mT: approximately 3 orders of magnitude lower than clinical intensities). Therefore, although our results suggest LI-rTMS does not promote cell survival or regeneration, it is possible that high intensity stimulation may have more powerful effects.

The impact of stimulation intensity on neural repair can be directly observed in two studies using rTMS or LI-rTMS in the same rat model of ischaemic stroke. One week of stimulation using a human coil resulted in a significant decrease in the lesion size after ischaemic stroke and improvement in motor behaviour [17]. By contrast, stimulation with LI-rTMS at the same frequency, although for a shorter duration, did not result in similar benefits [37]. The importance of stimulation intensity was further highlighted in studies of direct electrical stimulation, in which stimulation with 30 to 70μA, but not 20μA increased RGC survival following an optic nerve transection [24]. Consistent with the different outcomes, there is evidence that rTMS and LI-rTMS activate different mechanisms: high intensity rTMS elicits activation of neural circuits via synaptic plasticity [8, 38] whereas LI-rTMS is subthreshold and exerts its effects by altering membrane potential and neuronal intracellular calcium concentrations without eliciting action potentials [31]. Therefore it may be that lower intensities are sufficient to promote circuit reorganisation in intact tissue but higher intensities are required for neuroprotection.

### LI-rTMS effects may be brain region specific

The cellular mechanisms activated by rTMS and LI-rTMS are poorly understood, but one factor that is commonly detected is up-regulation of BDNF, regardless of the intensity of stimulation[10–13, 39]. Our previous work showed that LI-rTMS using the protocol applied here induces structural plasticity and up-regulates BDNF in multiple visual brain centres [10, 11] and in the lesioned olivocerebellar pathway [26]. However, in the present study, the same LI-rTMS protocol delivered directly to the eye did not significantly alter BDNF in the retina or optic nerve following 7 days of stimulation. We chose this time-point because of the dynamics of RGC death in our model. Following optic nerve crush, approximately 50% of RGCs survive at one week [22, 40], whereas less than 10% remained at 2 weeks. Measuring BDNF at 1 week therefore maximises the chances of detecting changes because the low survival rate at 2 weeks makes a delayed up-regulation unlikely. Nonetheless, we cannot exclude the possibility that up-regulation might have occurred before or after this time.

A possible explanation for the lack of BDNF up-regulation is that the rapid death of RGCs and/or complete discontinuity between the retina and brain targets in our model prevented the effects of LI-rTMS. Supporting this possibility, transcranial alternating current stimulation (tACS) induced EEG after-effects in intact rats but not in animals with severe optic nerve damage [41] and the authors suggested that their finding of 9% RGC survival following optic nerve damage was below the threshold needed for tACS to have an effect. A similar problem may apply to our complete optic nerve crush model (<10% RGC survival), whereby LI-rTMS failed to induce positive effects due to too few surviving RGCs and the lack of connections to central targets. This hypothesis is in agreement with our previous studies showing that LI-rTMS promotes beneficial reorganisation of *existing* connections [11, 26]. Therefore less severe neurotrauma models may respond more effectively to LI-rTMS. Furthermore, partial lesion models [42, 43] may help to determine if a minimum proportion of surviving RGCs and central connections are needed to provide a substrate for LI-rTMS to promote survival and beneficial reorganisation of spared connections.

A further consideration is that the retinofugal pathway lacks the complex excitatory and inhibitory circuitry of the cerebral cortex upon which rTMS is thought to act [7, 8, 44] However, we stimulated the retina, which possesses complex regulatory inhibitory and excitatory circuits that have been compared to those in the cortex [45] and it may therefore provide an appropriate substrate for rTMS if the relevant protocols and models are established. In addition to exploring the role of magnetic field intensity, it will be therefore be important to examine the effect of stimulation frequencies and number of pulses on cell survival and regeneration due to possible frequency and dose-dependent effects of rTMS on complex circuitry [46–48].

### Non-invasive brain stimulation techniques for treating neurotrauma

Our result that chronic LI-rTMS does not increase cell survival at 2 weeks is similar to previous reports of other types of non-invasive brain stimulation interventions following complete optic nerve crush injury. For example, tACS stimulation failed to increase RGC survival in the acute phase (1 week post injury) but RGC survival was improved compared to controls at 4 weeks post crush [22]. The authors suggest tACS may act upon the delayed mechanisms of retrograde cell death. By contrast, another study found that transcorneal electrical stimulation (TES) resulted in increased RGC survival at 1 week post optic nerve crush but this was not sustained at 2 weeks [23]. These results suggest that different non-invasive brain stimulation methods may have diverse mechanisms of action that are effective at different times in the cell death cascade (early vs. late phase). In one study, rTMS was delivered in the acute post stroke phase (1 hour post stroke) and found decreased apoptosis and improved function [49]. However, stimulation within 24 hours of an injury may be inappropriate, due to abnormal excitability observed in the acute stages post trauma [50, 51]. Yoon and colleagues examined the use of rTMS in the sub-acute stage (4 days post stroke) and found an increase in anti-apoptotic proteins with improved behavioural function [52]. Therefore, although our results suggest that LI-rTMS is not neuroprotective in the acute phase post injury, the impact of LI-rTMS on later stages of retrograde cell death should be explored, perhaps in combination with other types of electrical stimulation applied acutely following injury (e.g. TES).

## Conclusion

As the mechanisms of action of non-invasive brain stimulation techniques become increasingly well understood through human and animal studies, it is important to continue to explore the potential for previously unconsidered therapeutic effects. The disappointing outcomes for neuroprotection and regeneration following LI-rTMS relative to other non-invasive brain stimulation protocols suggest that LI-rTMS is not adapted to this purpose. Rather LI-rTMS may better be applied to aid neural rehabilitation by modulating the plasticity of spared tissue after injury [53]. Furthermore, our current and previous work suggests that LI-rTMS may still have a role in the protection and conservation of *intact* RGCs after trauma, as long as these neurons retain the ability to retrogradely transport BDNF after injury. In addition to exploring the role of magnetic field intensity, it will be important to examine the effect of stimulation frequencies and number of pulses on cell survival and regeneration due to possible frequency and dose-dependent effects of rTMS [46–48]. In summary, the results from this study help define the therapeutic utility and scope of LI-rTMS treatment and suggest that although LI-rTMS can induce plasticity in intact tissue, it does not induce neuroprotection immediately after severe neurotrauma. Future studies should examine the application of LI-rTMS in more acute and sub-acute stages following neurotrauma and in less severe injury models such as partial lesions.
